# The altered activity of P53 signaling pathway by *STK11* gene mutations and its cancer phenotype in Peutz-Jeghers syndrome

**DOI:** 10.1186/s12881-018-0626-5

**Published:** 2018-08-09

**Authors:** Yu-Liang Jiang, Zi-Ye Zhao, Bai-Rong Li, Fu Yang, Jing Li, Xiao-Wei Jin, Hao Wang, En-Da Yu, Shu-Han Sun, Shou-Bin Ning

**Affiliations:** 1 Hebei North University, Zhangjiakou, 075061 Hebei Province China; 2 Department of Gastroenterology, Airforce General Hospital of PLA, Beijing, 100142 China; 3 Department of Medical Genetics, Naval Medical University, Shanghai, 200433 China; 4 Department of Colorectal Surgery, Changhai Hospital, Shanghai, 200433 China

**Keywords:** Peutz-Jeghers syndrome (PJS), *STK11* gene, *P53* gene, Cancer risk, Reporter gene technique

## Abstract

**Background:**

Peutz-Jeghers syndrome (PJS) is caused by mutations in serine/threonine kinase 11 (*STK11*) gene. The increased cancer risk has been connected to P53 pathway.

**Methods:**

PJS probands with *STK11* mutation were included in the function analysis. P53 activity elevated by *STK11* mutants was investigated using dual-luciferase reporter assay in vitro after constructing expression vectors of *STK11* wild type and mutants generated by site-directed substitution. The association between the P53 activity and clinicopathological factors was analysis, especially the cancer history.

**Results:**

Thirteen probands with *STK11* mutations were involved, and within the mutations, c.G924A was novel. P53 activity elevation caused by 6 truncating mutations were significantly lower than that of *STK11* wild type (*P* < 0.05). Family history of cancer was observed in 5 families. Within them, P53 activity was reduced and cancer occurred before 40 in 2 families, while it was not significantly changed and cancers happened after 45 in the other 3 families.

**Conclusions:**

The affected P53 activity caused by *STK11* mutations in PJS patients is significantly associated with protein truncation, while cancer risk in PJS can be elevated through pathways rather than P53 pathway. P53 activity test is probably a useful supporting method to predict cancer risk in PJS, which could be helpful in clinical practice.

**Electronic supplementary material:**

The online version of this article (10.1186/s12881-018-0626-5) contains supplementary material, which is available to authorized users.

## Backgroud

Peutz-Jeghers syndrome (PJS; OMIM #175200) is an autosomal dominant disorder characterised by mucocutaneous melanin pigmentation (MP), gastrointestinal(GI) hamartomatous polyposis, and an increased risk for the development of various neoplasms [1, 2], which is rare with a low incidence of 1/50,000 [3]. The cumulative lifetime risk is 20, 43, 71, and 89% at ages of 40, 50, 60 and 65 years, respectively [4].

Germline mutations in the serine–threonine kinase 11 (*STK11*) gene on chromosome 19p13.3 were identified as a major genetic cause of PJS in 1998 [5, 6]. This gene is divided into 9 coding exons that encode a 433 amino-acid protein, which acts as a tumor suppressor. The STK11 protein is mainly comprised of 3 major domains: an N-terminal regulatory domain, a catalytic kinase domain, and a C terminal regulatory domain [7]. Amino acids 49–309 of STK11 are the catalytic kinase domain, which can form a complex with STRAD and scaffold protein 25 (MO25) to maintain kinase activation [8]. Several studies have described a role of STK11 in cell cycle arrest [9], P53 mediated apoptosis [10], Wnt signaling [11, 12], TGF-β signaling [13], Ras induced cell transformation [14], and cell polarity [15–18].

*P53*, the most widely studied tumor suppressor gene, regulates of the expression of various genes that are related to cell cycle arrest, DNA damage repair and apoptosis [19], and furtherly controls the cell proliferation and apoptosis. *P53* gene mutations have been found in about 50% of human cancers, suggesting the important role of *P53* inactivation in tumorigenesis [20]. *STK11* has been reported to play a critical role in P53-mediated cell apoptosis [10]. *STK11*^+/−^/*P53*^+/−^ mice showed a dramatically reduced life span and increased tumor incidence compared to the mice with either *STK11* or *P53* single gene knockout, indicating that *P53* and *STK11* gene mutations cooperate in tumor progression [21].

In this study, we identified mutations in *STK11* gene from Chinese PJS probands, and observed the changes in P53 activity brought by different *STK11* mutants and their association with the canceration in PJS.

## Methods

### Patient and sample collection

Materials in this study were collected retrospectively. A total of 154 PJS patients were ascertained in Airforce General Hospital of PLA between May 2013 and April 2016. Blood samples of the probands and all available family members were collected after obtaining informed consent. This study was approved by the Medical Ethics Committee, Airforce General Hospital of PLA. Only PJS patients with both complete information and mutation detected and successfully constructed were included in final analysis.

Clinical diagnosis for PJS was based on the presence of any one of the following clinical findings which was recommended by WHO [22]: (1) three or more histologically confirmed PJ polyps (PJP) in the gastrointestinal tract, (2) any number of PJPs detected with a positive family history of PJS, (3) characteristic mucocutaneous pigmentation with a positive family history of PJS, and (4) any number of PJPs together with characteristic mucocutaneous pigmentation (MP).

### Genomic DNA isolation and mutation analysis

Genomic DNA of peripheral blood leucocytes was extracted routinely by animal genomic DNA kit (TSP201, TsingKe Biotech, Beijing, China) according to the manufacturer’s instructions. All 9 coding exons and their flanking sequences of the *STK11* gene were amplified by the use of primers listed in Additional file 1: Table. S1. Polymerase chain reactions (PCR) of *STK11* exons were performed in a 50-ul reaction which contained 0.4 uM of each primer, 50 ng genomic DNA, and 25ul 2 × modified DNA polymerase mix (TSE004, TsingKe Biotech, Beijing, China). The PCRs were performed under the following conditions: denaturation at 95 °C for 4 min, followed by 35 thermal cycles, each composed of 95 °C for 30 s, 58 °C for 30 s, and 72 °C for 45 s.

All available family members underwent *STK11* germline mutation test to confirm cosegregation of the mutation with the disease. For frameshift mutation, T vector assay (pClone007 Vector Kit, TSV-007, TsingKe Biotech, Beijing, China) was used to identify each haplotype by constructing monoclonal cells [23]. In order to rule out polymorphisms and to confirm the pathogenic effects of the variations, 100 chromosomes from 50 unrelated ethnicity-matched healthy individuals were also screened for the presence of the mutations. Allele frequency of each mutation in ExAC was checked [24].

The PCR products were gel- and column-purified and directly sequenced. The purified PCR fragments were then sequenced using BigDye Terminator on an ABI Prism 3100 genetic analyzer (Applied Biosystems, Foster City, CA, USA) by Majorbio Co. Ltd. (Shanghai, China). The results were used to performance sequence alignment according to *STK11* gene sequence (NP_000446.1 and NM_000455.4 in GRCh38.p7). All experiment details above had been reported previously [23, 25].

### Function prediction and analysis, and association with Clinicopathological features

To predict the effects of mutations in *STK11*, PolyPhen-2 (http://genetics.bwh.harvard.edu/pph2) and SIFT (http://sift.jcvi.org/) were used for specific mutations.

To investigate the impact on P53 activity, we employed dual-luciferase reporter gene technique. Human *STK11* cDNA was amplified from the HEK293T cells, and cloned into pcDNA3.1 (+) (V790–20, Carlsbad, CA) vector to create expression vectors of wild type (wt) and mutant (mu) of *STK11* gene. The mutant site was generated by site-directed substitution (D0206, Beyotime, Nantong, Jiangsu, China).

The luciferase reporter plasmid pp53-TA-luc which contained a P53-responsive element (D2223, Beyotime, Nantong, Jiangsu, China) was transfected into the cells, together with pRL-TK vector (E2241, Promega, Madison, WI) and pcDNA3.1(+)-*STK11* expression vector, using the Lipo6000 transfection reagent (C0526, Beyotime, Nantong, Jiangsu, China). pRL-TK vector expresses wildtype Renilla luciferase reporter and is used as internal control by cotransfected with pp53-TA-luc. Twenty-four hours after transfection, cells were lysed in a passive lysis buffer, which was a component of Dual-Luciferase® Reporter Assay Systems (E1910, Promega, Madison, WI) [26]. According to the manufacturer’s protocol, luciferase activity was measured with the Synergy 2 modular multi-mode reader (BioTek Instruments, Winooski, VT). The ratio of pp53 to pRL-TK was calculated in each assay. Each experiment was repeated triple times from cell seeding to luciferase activity measurement. Data were showed as mean ± standard deviation. Student’s t test was used to test the significance, and *p* < 0.05 was considered as significant.

Factors related to cancer history including sex, family history and P53 activity change were compared using the Fisher exact test, and *p* < 0.05 was considered as significant.

## Results

During the period investigated, 154 PJS patients were diagnosed in our department, among whom, 89 declined to participant in mutation test, and 26 had inadequate information. Finally, 39 patients from 31 unrelated families had blood samples collected and received mutation detection. A total of 17 disease-specific mutations in *STK11* were detected in 31 probands. Since 3 mutations concerning splicing site (c.290 + 1G > A, c.734-1G > A, c.863-8_870del16bp) could not be investigated by reporter gene assay, and one mutation (c.426_448del23bp) was hard to be generated by site-directed mutagenesis, there were 13 mutants involved in the final analysis (Additional file 2: Figure S1). Expression vectors of the wild-type and the 13 mutant *STK11* gene were constructed for function analysis, and the corresponding PJS families and individuals were included in analysis.

### Clinical features

**Fig. 1:**
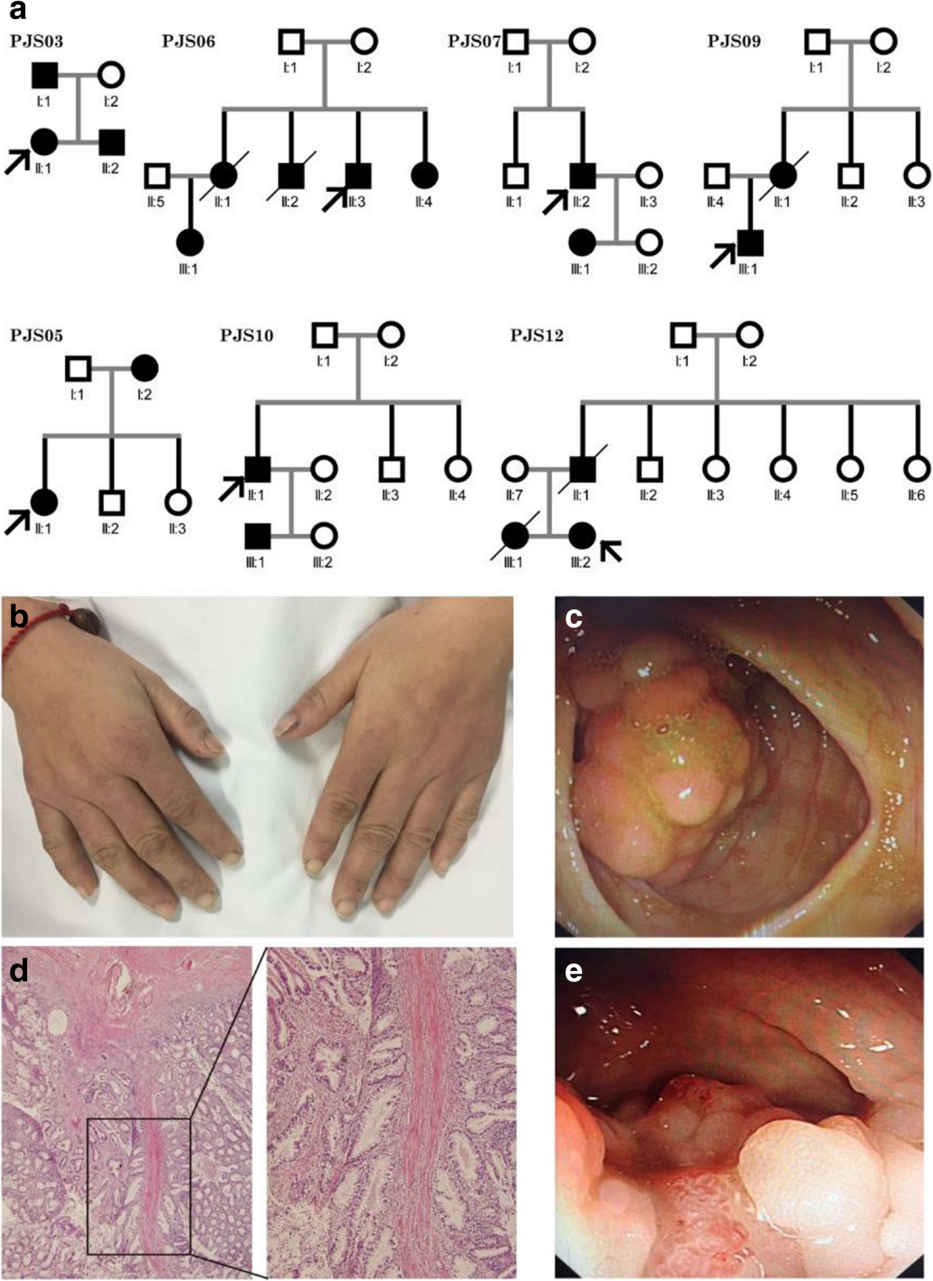
Genogram and Clinicopathological feature of PJS patients. **a** Pedigrees of the 7 PJS cases with positive family history. Roman numerals indicate generations and Arabic numbers indicate individuals. Squares = males, circles = females. Affected individuals are denoted by solid symbols and unaffected individuals are denoted by open symbols. The probands are indicated by arrows. **b** and **c**. Melanin pigmentation spots of the fingers, endoscopic view of the recurrent polyp near the anastomotic stoma after the radical surgery of colon cancer and were observed in the proband of PJS03. **d** Hematoxylin-eosin-stained tissue slices of the polyp specimens above confirm hamartomatous. Left, × 40 magnification; right, × 100 magnification. **e** Endoscopic view of the polyps in PJS11, one of which had cancerogenesis

As shown in Table 1, the 13 probands from unrelated families were 7 familial and 6 sporadic patients, and their native places were various in China (Additional file 1: Table S2). The median age of diagnosis, first polypectomy and at present was 25, 28 and 33 years. Seven of these families (PJS03, 05, 06, 07, 09, 10 and 12) showed autosomal dominant pattern and the remaining 6 cases (PJS01, 02, 04, 08, 11 and 13) are sporadic (Fig. 1a). MP on the lips, buccal mucosa, and digits were observed at all patients (Fig. 1b). Twelve probands in these families underwent laparotomy or polypectomy due to intussusception or intestinal obstruction (Fig. 1c and d). Three probands of these families (PJS03, 06 and 11) in this study had developed colon cancer before 40 years old (Fig. 1e), and cancer history existed in 3 families (PJS06, 09 and 12). Detail information is showed in Table 2.

**Table 1:** Characteristics of the cohort with PJS

Characteristics		Number (percentage)	
Probands with mutation test		31	
Mutation detected		17	54.8%
Probands enrolled in the luciferase analysis		13	
Age^a^ Median (range)	D	25 (12–37) years	
	F	28 (11–39) years	
	P	33 (24–51) years	
Gender			
Male		7	51.8%
Female		6	48.2%
Family history			
Familial		7	51.8%
Sporadic		6	48.2%
Cancer history		5	38.5%

^a^ D, diagnosis; F, first polypectomy or laparotomy; P, present. There were 12 probands having polypectomy

**Table 2:** Clinical characteristics and function analysis of *STK11* mutations in PJS patients

ID	FH^a^	Sex^b^	Age^c^			Polyp location	Int	Mutation	Exon	Function domain	Effect	Function Prediction		ExAC allele frequency	P53 activity	Index cancer	Cancers in family
			D	F	P							PolyPhen-2	SIFT				
PJS01	S	M	12	11	24	SB, C	Yes	c.56C > G	1	N	p.S19 W	0.663	0.00	0	Normal		
PJS02	S	F	15	15	29	SB	Yes	c.193G > T	1	I	p.E65^a^	NA	NA	0	Decreased		
PJS03	F	F	31	27	33	SB, C	Yes	c.250A > T	1	II	p.K84^a^	NA	NA	0	Decreased	colon	
PJS04	S	F	17	12	28	SB	Yes	c.354C > G	2	IV	p.Y118^a^	NA	NA	0	Decreased		
PJS05	F	F	23	23	27	SB, C	Yes	c.842delC	6	XI	p.P281Rfs^a^6	NA	NA	0	Decreased		
PJS06	F	M	21	21	45	SB, C	Yes	c.843insC	6	XI	p.P281Pfs^a^4	NA	NA	0	Decreased	colon	lung, lymphoma
PJS07	F	M	32	32	38	SB, C	Yes	c.855delG	6	XI	p.L285Lfs^a^2	NA	NA	0	Decreased		
PJS08	S	F	31	15	35	G, SB, C	Yes	c.862G > A	6	XI	p.G288S	0.0.573	0.10	0	Normal		
PJS09	F	M	30	30	51	SB, C	No	c.911G > C	7	XI	p.R304P	0.0.987	0.05	0	Normal		stomach
PJS10	F	M	24	39	43	SB, C	Yes	c.924G > A	8	XI	p.W308^a^	NA	NA	0	Normal		
PJS11	S	M	37	37	47	C	Yes	c.962_963delCC	8	C	p.P321Hfs^a^38	NA	NA	0	Normal	colon	
PJS12	F	F	12	12	29	G, SB, C	Yes	c.1062C > G	8	C	p.F354 L	0.022	0.36	0.005757	Normal		colon
PJS13	S	M	24	23	30	G	Yes	c.1225C > T	9	C	p.R409W	0.549	0.00	0.000147	Normal		

^a^Family history. S = sporadic, F = familial. ^b^ M = male, F = female. ^c^D, diagnosis; F, first polypectomy or laparotomy; P, present. If a patient is dead, the present age with a underline refers to the age of death. G = stomach, SB = small bowel, C = colon. Int = intussusception. NA = not available

### Mutation analysis

**Fig. 2:**
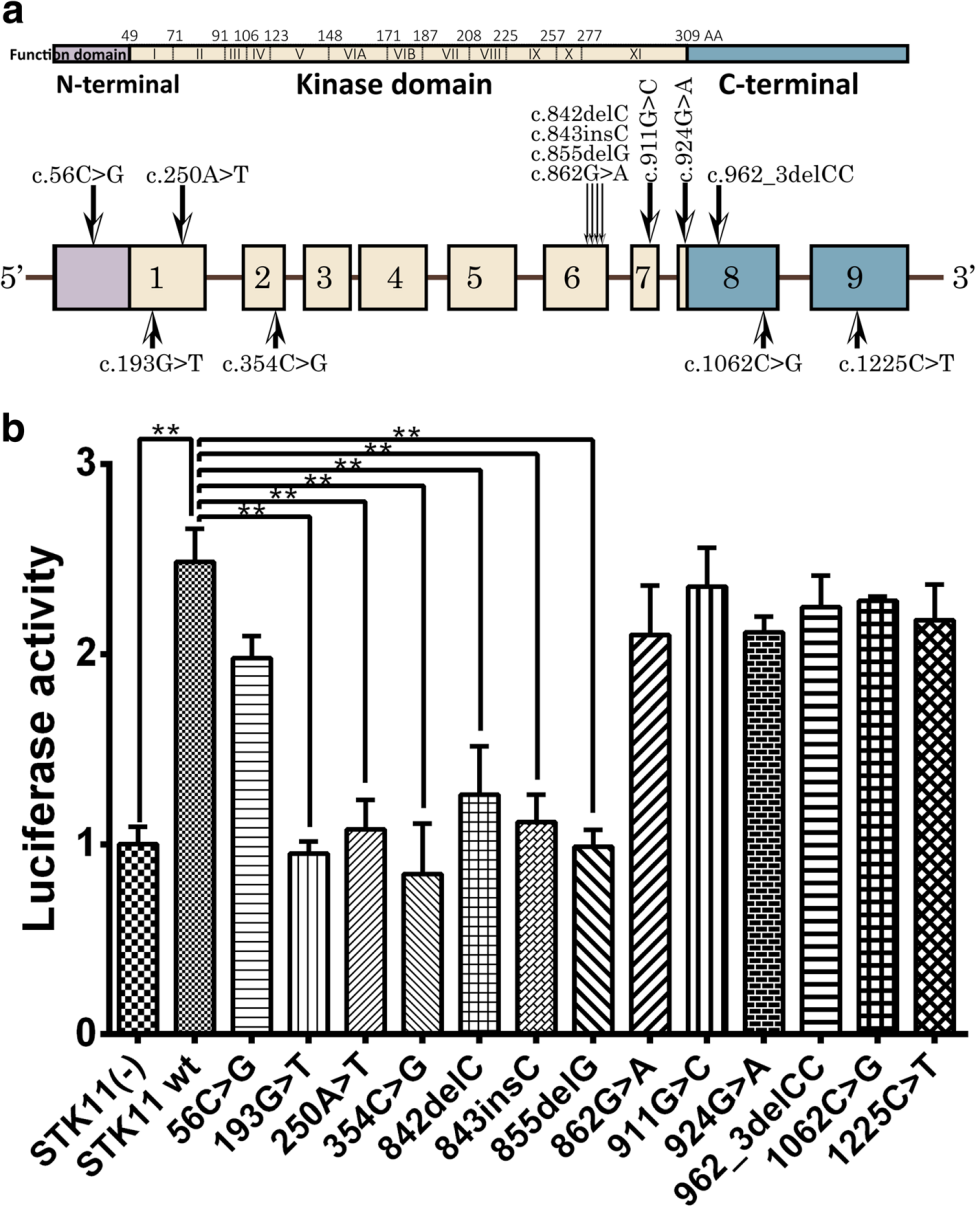
Distribution of all the *STK11* mutations detected (**a**) and P53 pathway activity tested by dual-luciferase reporter gene technique in HEK293T cells transfected with these mutants (3 replicates) (**b**). Double-asterisks present significant (*p* < 0.05)

**Fig. 3:**
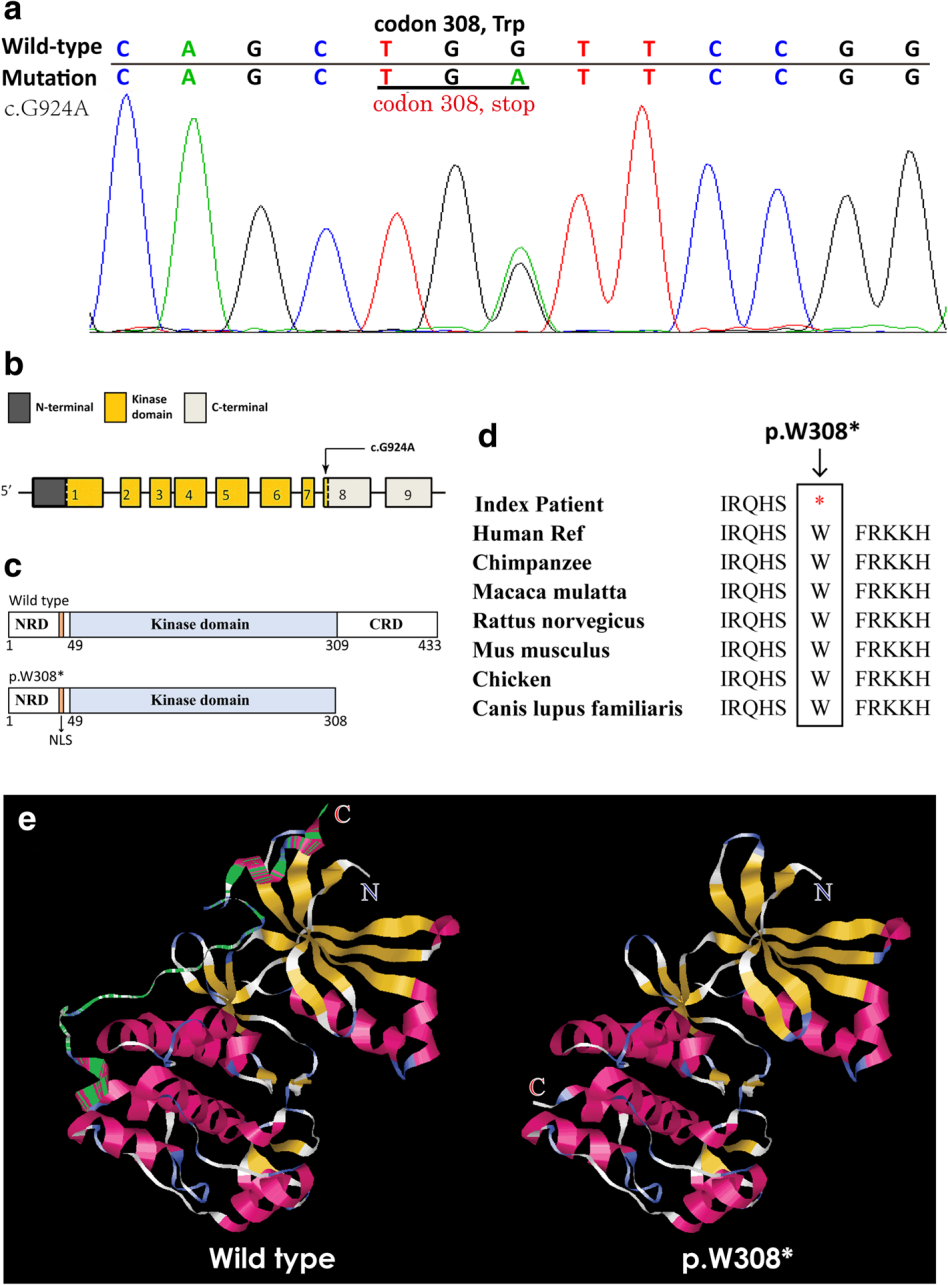
Details about the novel mutation c.G924A (p.W308*). **a** Sanger sequencing showed a heterozygous mutation. **b** The structure of STK11 gene. The mutation is located in exon 8. **c** Schematics of the secondary structure or functional domains of the STK11 protein. The mutant protein results in a partial loss of kinase domain and a complete loss of the C-terminal domain compared to the wild type. NLS, Nuclear localization signal, NRD or CRD, N- or C-terminal regulatory domain. **d** Evolutionary conservation of amino acid residues altered across different species. **e** The mutant proteins was predicted to result in partial loss of the kinase domain and complete loss of the C-terminal domain of the a-helix (which is labelled using green gaps in the wild type protein) by Swiss-Model online software (http://swissmodel.expasy.org/) compared to the wild type (for which the 3D template model used was 2wtk.2.C) [46]

Sequencing analysis identified 13 mutations of *STK11* gene in the 13 probands which were included in the function analysis (Table 2, Fig. 2a). Most of the mutations have been previously reported [25, 27–29] except for c.924G > A (Fig. 3). The 13 mutations included 8 truncating mutations (4 frameshift and 4 nonsense) and 5 missense mutations. Regarding frameshift mutations, those of PJS05, 06 and 07 (p.P281Rfs*6, p.P281Pfs*4 and p.L285Lfs*2) led to a partial loss of the kinase domain and a completed loss of the C-terminal, and the one in PJS11 (p.P321Hfs*38) led to a partial loss of the α-helix of the C-terminus. PJS02, 03, 04 and 10 possessed nonsense mutations of *STK11* that resulted in the production of truncated proteins (p.E65*, p.K84*, p.Y118* and p.W308*) (Additional file 2: Figure S2A). Among the PJS cases owning the 13 mutations, 7 were familial and 6 were sporadic, and 5 of them were associated with cancer history. Tested in 100 chromosomes from control individuals, 16 of the detected mutations were not discovered and only c.1062C > G was detected in 5/100 controls. c.1062C > G and c.1225C > T were recorded in ExAC database with allele frequencies of 0.005757 and 0.000147, and all other mutations were not recorded (Table 2).

### Function analysis and association with Clinicopathological features

Analyzed using PolyPhen-2, missense mutation c.911 was predicted as probably damaging, mutations c.56, c.862 and c.1225 were predicted as possible damaging, while c.1062 was benign (Table 2).

Analyzed using SIFT, missense mutation c.56 and c.1225 were predicted as intolerated, and mutations c.862, c.911 and c.1062 were tolerated (Table 2).

P53 activity was measured using dual-luciferase reporter gene technique after cotransfection of pp53-TA-luc, pRL-TK vector, and pcDNA3.1 (+)-*STK11*-wt or -mu. Luciferase activity was significantly elevated when *STK11*-wt was transfected into HEK293T cells. Using the elevation caused by *STK11*-wt as control, elevations by mutants c.193, c.250, c.354, c.842, c.843 and c.855 were significantly reduced, while those by mutants c.56, c.862, c.911, c.924, c.962_963, c.1062 and c.1225 were not changed significantly (Fig. 2b).

There were total 5 families with cancer history (PJS03, 06, 09, 11 and 12). Within the 5 cancer families, P53 activity was reduced and cancer occurred before 40 in 2 families (PJS03 and 06), while in the other 3 families, P53 activity was not significantly changed and cancers happened after 45 years old (PJS09, 11 and 12) (Table 3).

**Table 3:** Characteristics of affected individuals in PJS families with cancer history

Family	Individual	Sex	Age^b^					Cause of death	Mutation	Prediction		P53 activity
			MP	D	F	C	P			PolyPhen	SIFT	
PJS03	I:1	M	NR	NR	NR	60	60	Colon cancer	c.250A > T/ p.K84^a^	NA	NA	Decreased
	II:1^a^	F	5	31	27	27	33	Colon cancer				
	II:2	M	6	19	26	–	28	–				
PJS06	II:1	F	NR	NR	NR	NR	40	Lung cancer	c.843insC/ p.P281Pfs^a^4	NA	NA	Decreased
	II:2	M	NR	NR	NR	NR	39	Lymphoma				
	II:3^a^	M	3	21	21	39	45	Colon cancer				
	II:4	F	3	20	20	–	39	–				
	III:1	F	3	11	18	–	30	–				
PJS09	II:1	F	NR	55	NR	65	65	Stomach cancer	c.911G > C/ p.R304P	Probable damaging	Tolerated	Normal
	III:1^a^	M	7	30	30	–	51	–				
PJS11	Index^a^	M	6	37	37	47	47	Colon cancer	c.962_963delCC/ p.P321Hfs^a^38	NA	NA	Normal
PJS12	II:1	M	NR	43	43	48	52	Colon cancer	c.1062C > G/ p.F354 L	Benign	Tolerated	Normal
	III:1	F	4	15	–	–	16	Bowel obstruction				
	III:2^a^	F	4	12	12	–	29	–				

^a^ Probands. ^b^ MP, melanin pigmentation; D, diagnosis; F, first polypectomy or laparotomy; C, canceration; P, present. If a patient is dead, the present age with a underline refers to the age of death. M = male, F = female. NA = not applicable. NR = not reported

Through Fisher exact test, none of the factors (sex, family history and P53 activity change) was significantly associated with cancer in PJS (Additional file 1: Table S3).

## Discussion

Peutz-Jeghers syndrome (PJS) has various manifestations related to GI polyps, such as abdominal pain, rectal blood loss, chronic anemia, prolapsed rectal polyp, bowel obstruction and clinical intussusception [30–32]. Beyond these symptoms, PJS patients also have an increased risk of cancer at multiple sites, including the GI tract, breast, ovary, testis, and lung [33]. As the only validated and the major pathogenic gene in PJS, *STK11* is involved in cell cycle regulation and apoptosis, whose abnormality can induce and promote tumorigenesis. It has been proven that *STK11*-mediated cell cycle regulation has been shown to be regulated via P53-dependent and P53-independent mechanisms [34–39]. STK11 can directly interact with and phosphorylate P53, and to mediate P53-induced apoptosis [40]. What’s more, PJS-associated *STK11* mutants can diminish P53 activity [41].

In our study, we investigated the effect of *STK11* mutations on the transcriptional activity of P53, and we found that six of them inhibited the activity significantly compared with the wild-type. All of the 6 mutations resulted in protein truncation, and all the truncated protein lost partial kinase domain and completed C-terminal. The other seven mutations which didn’t significantly decrease P53 activity located in the margin of or outside the kinase domain, and they caused only single amino acid change in the margin of or outside the kinase domain or loss of C-terminal regulatory domain. So we suspected the main part of the kinase domain or some key points within it was key for STK11 depended P53 activity rather than the boundary of it or regulatory parts, and truncating mutations were easier to cause the change since they usually resulted in large-scale loss of the amino acid residues. We also encountered three splice site mutations, which cannot be investigated by the luciferase assay. Since splicing errors usually cause exon skipping or abnormal mRNA processing and mRNA degradation, the large-scale changes may also result in P53 activity decrease. But due to the limitation of patient number, the assumption cannot be justified at the present.

As to the association between P53 activity reduction and cancerogenesis, there is no significance under statistical analysis. One of the reasons is the limited number of cases involved in analysis, just like another two analyses (sex and family history) without statistical significance. When analysed one by one, we can discover some implications from them. Early onset is a distinguishing feature of inherited cancer, and among our cases, there are cancers patients younger than 40 in PJS03 and 06 whose P53 activity is reduced by their mutations. While in PJS09, 11 and 12, cancer patients are older than 45, which means they are more like sporadic ones. Though there is a death case of youth in PJS12, it is not a certain case of cancer. More important, the mutation c.1062C > G is now regarded as a benign variation according to guideline from American College of Medical Genetics and Genomics (ACMG) [42, 43]. So most likely there is a large scale deletion rather than point mutation like c.1062C > G which is to blame. Another possibility is that P53 pathway is not the key one taking part in these three PJS families’ cancerogenesis, and some non-P53 pathways leads to the occurrence of tumors.

There are still four families without cancer diagnosed, whose mutations were tested to impact P53 activity. The four mutation carriers are still young (29, 28, 27 and 38 years), so it is important to carry out a more rigorous and comprehensive follow-up as cancer prevention before any advanced methods come up. As to the other four PJS families carrying *STK11* mutations without significant P53 activity change, routine surveillance is still necessary to keep them uneventful. The recommendations for PJS management produced by Mallorca conference 2007 is a comprehensive and widely accepted one, which we can refer to [44, 45].

There are several important limitations to note. First, here we try to elucidate the pathways of tumorigenesis in PJS by investigating the P53 activity change caused by *STK11* mutations, but the mutations are not evenly distributed within the coding sequence and the limited number of cases involved largely limits the results. Second, quite a few of *STK11* mutations in PJS are large fragment deletion, so it is necessary to perform test like multiplex ligation–dependent probe amplification (MLPA) assay to make a more comprehensive analysis in the future. Finally, this is a single center study, and the data here may not be the whole picture of PJS in China. It will be very helpful if there is a national database of PJS patients.

## Conclusions

The affected P53 activity caused by STK11 mutations in PJS patients is significantly associated with protein truncation, while cancer risk in PJS can be elevated through pathways rather than P53 pathway. P53 activity test is probably a useful supporting method to predict cancer risk in PJS, which could be helpful in clinical practice.

## Additional files

Additional file 1: **Table S1**. Primers for exon-specific sequencing of *STK11* gene. **Table S2**. Native place of PJS patients. **Table S3**. Association between cancer history and certain factors in the cohort of PJS. (DOCX 24 kb)

Additional file 2: **Figure S1**. Flow diagram of patients enrollment. PJS indicates Peutz-Jeghers syndrome. **Figue S2**. The predicted protein structure of 8 truncating mutations (A) and the PolyPhen-2 prediction results for 5 missense mutations (B). NLS, nuclear location signal. **Figure S3**. Western blot results of the mutation investigated. Targeted protein: STK11; internal reference: GAPDH. (ZIP 2634 kb)

## Abbreviations

DBE: Double-balloon enteroscopy
GI: Gastrointestinal
MLPA: Multiplex ligation–dependent probe amplification
MP: Melanin pigmentation
PCR: Polymerase chain reaction
PJP: Peutz-Jeghers syndrome polyp
PJS: Peutz-Jeghers syndrome
*STK11*: Serine/threonine kinase 11
