# Supplementary material for: The altered activity of P53 signaling pathway by STK11 gene mutations and its cancer phenotype in Peutz-Jeghers syndrome
Source: BMC Med Genet. 2018 Aug 9;19:141. doi: 10.1186/s12881-018-0626-5 (PMC6085611; doi:10.1186/s12881-018-0626-5)
Supplement: Supplementary file 1 — Table S1. Primers for exon-specific sequencing of STK11 gene. Table S2. Native place of PJS patients. Table S3. Association between cancer history and certain factors in the cohort of PJS. (DOCX 24 kb) [file 12881_2018_626_MOESM1_ESM.docx]

**Supplementary Tables**

| **Table S1. Primers for exon-specific sequencing of *STK11* gene** | | |
| --- | --- | --- |
| **Exon** | **Forward primer (5’-3’)** | **Reverse primer (5’-3’)** |
| 1 | CCGTTGGCACCCGTGACCTA | ACCATCAGCACCGTGACTGG |
| 2 | GGGCGGATCACAAGGTCA | AGGAGACGGGAAGAGGAGC |
| 3 | TGTGCCCAGAGCAAGAGC | GCAGAAGAATGGCGTGAACC |
| 4 & 5 | AGGAGACGGGAAGAGGAGC | TGAACCACCATCTGCCGTAT |
| 6 | TGACTGACCACGCCTTTCTT | TGAGGGACCTGGCAAACC |
| 7 | CAGGGTCTGTCAGGGTTGTCC | CCGTCCGCTGCTCTGTCTT |
| 8 | ACTGCTTCTGGGCGTTTGC | AGGTGGGCTGGAGGCTTT |
| 9 | GGTTCTGTGCTGGCATTTCG | GGCTCTGACGCTGGTGGAT |
| 10a | TGCCCAGGCTGACCTCTTC | CGATGGCGTTTCTCGTGTTTT |
| 10b | GGATTTGAGCTGTGGCTGTGAG | AACACCGTGACTGCCGACCT |

| **Table S2. Native place of PJS patients** | |
| --- | --- |
| **ID** | **Native place** |
| PJS01 | Sichuan, Southwest China |
| PJS02 | Anhui, East China |
| PJS03 | Anhui, East China |
| PJS04 | Guangxi, South China |
| PJS05 | Shaanxi, Northwest China |
| PJS06 | Shandong, East China |
| PJS07 | Fujian, East |
| PJS08 | Ningxia, Northwest China |
| PJS09 | Anhui, East China |
| PJS10 | Sichuan, Southwest China |
| PJS11 | Beijing, North China |
| PJS12 | Xinjiang, Northwest China |
| PJS13 | Anhui, East China |

| **Table S3. Association between cancer history and certain factors in the cohort of PJS** | | | |
| --- | --- | --- | --- |
| **Characteristics** | **Cancer history** | | ***p*** |
|  | **Yes** | **No** |  |
| Gender |  |  | 1.00 |
| Male | 4 | 3 |  |
| Female | 4 | 2 |  |
| Family history |  |  | 0.266 |
| Familial | 3 | 4 |  |
| Sporadic | 5 | 1 |  |
| P53 activity |  |  | 1.00 |
| Normal | 4 | 3 |  |
| Decreased | 4 | 2 |  |
