# Supplementary figures and images for: The altered activity of P53 signaling pathway by STK11 gene mutations and its cancer phenotype in Peutz-Jeghers syndrome
Source: BMC Med Genet. 2018 Aug 9;19:141. doi: 10.1186/s12881-018-0626-5 (PMC6085611; doi:10.1186/s12881-018-0626-5)

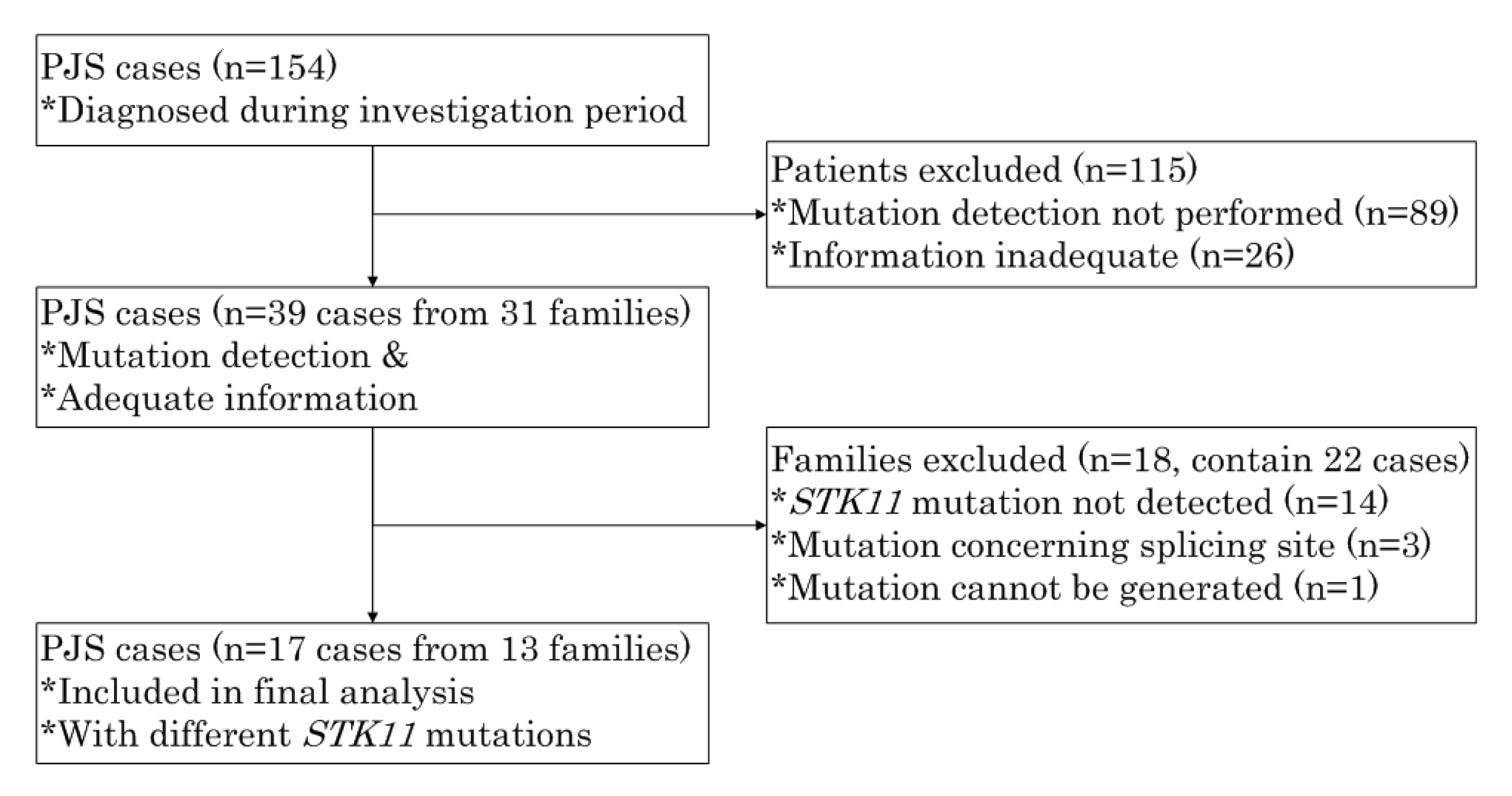

Supplement: Supplementary file 2 — Figure S1. Flow diagram of patients enrollment. PJS indicates Peutz-Jeghers syndrome. Figue S2. The predicted protein structure of 8 truncating mutations (A) and the PolyPhen-2 prediction results for 5 missense mutations (B). NLS, nuclear location signal. Figure S3. Western blot results of the mutation investigated. Targeted protein: STK11; internal reference: GAPDH. (ZIP 2634 kb) [file 12881_2018_626_MOESM2_ESM.zip › Fig S1R3.tif]

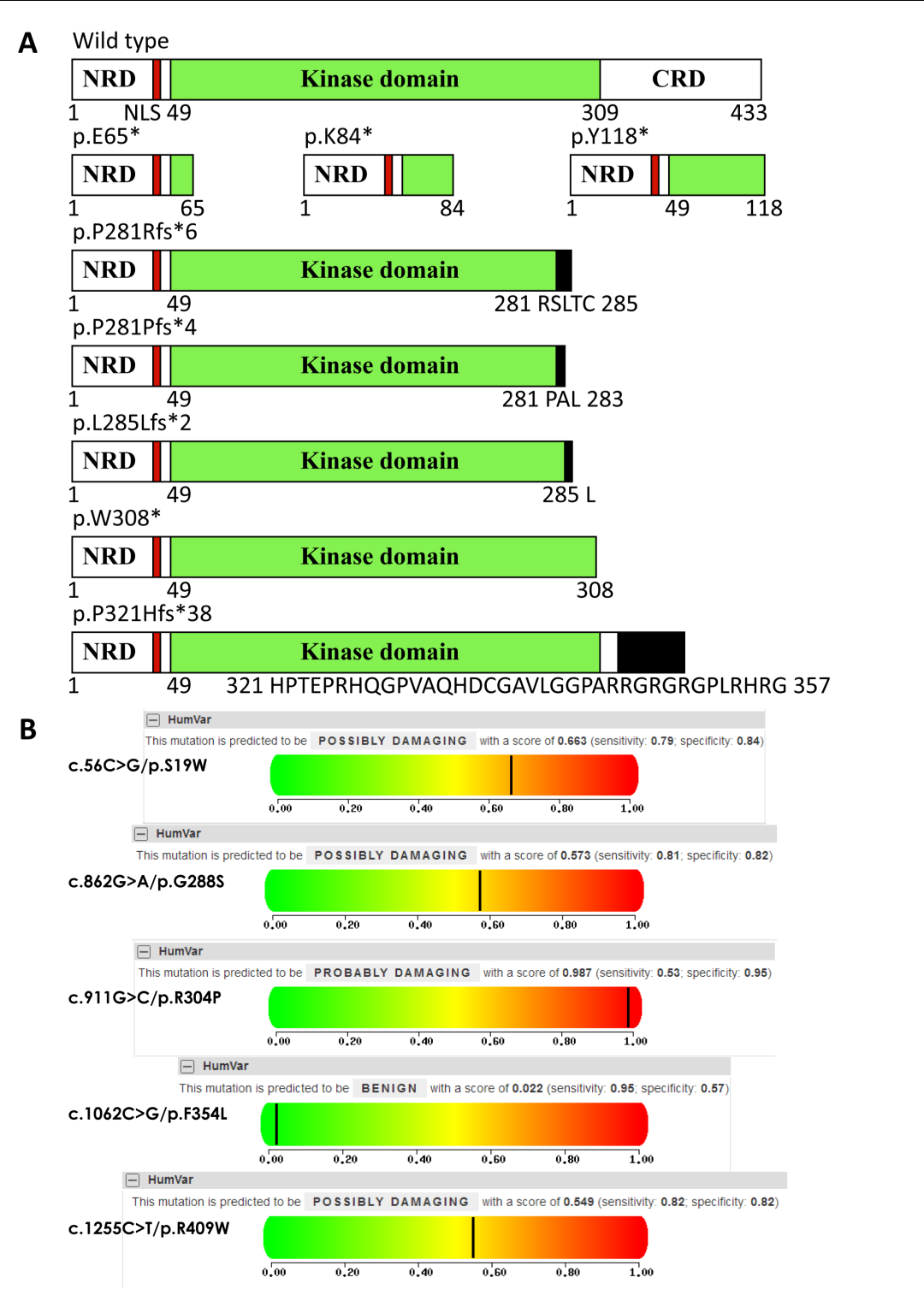

Supplement: Supplementary file 2 — Figure S1. Flow diagram of patients enrollment. PJS indicates Peutz-Jeghers syndrome. Figue S2. The predicted protein structure of 8 truncating mutations (A) and the PolyPhen-2 prediction results for 5 missense mutations (B). NLS, nuclear location signal. Figure S3. Western blot results of the mutation investigated. Targeted protein: STK11; internal reference: GAPDH. (ZIP 2634 kb) [file 12881_2018_626_MOESM2_ESM.zip › Fig S2R3.png]

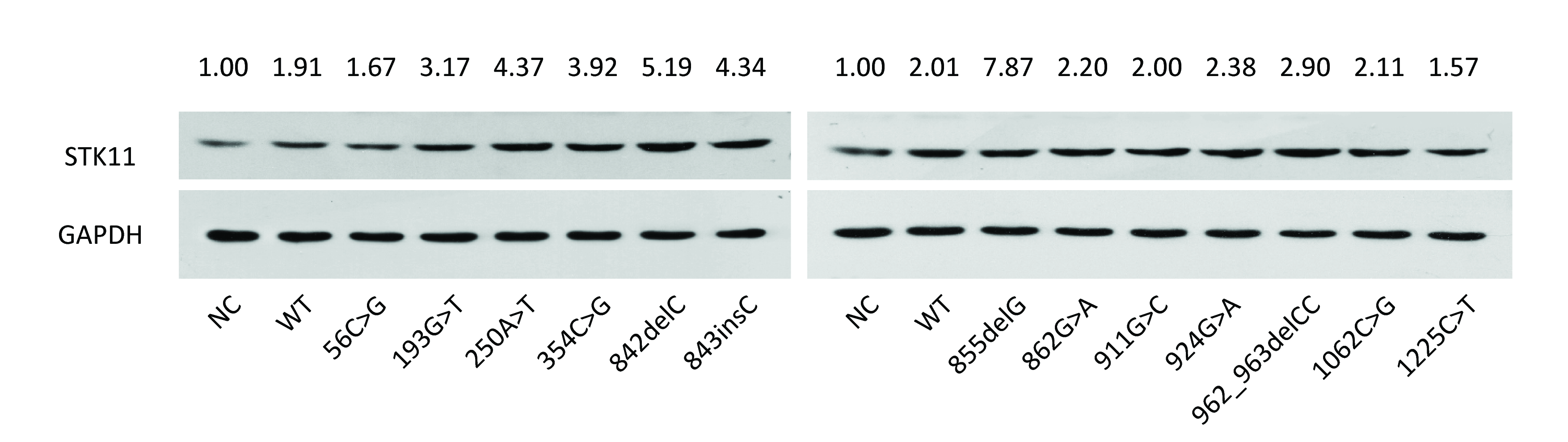

Supplement: Supplementary file 2 — Figure S1. Flow diagram of patients enrollment. PJS indicates Peutz-Jeghers syndrome. Figue S2. The predicted protein structure of 8 truncating mutations (A) and the PolyPhen-2 prediction results for 5 missense mutations (B). NLS, nuclear location signal. Figure S3. Western blot results of the mutation investigated. Targeted protein: STK11; internal reference: GAPDH. (ZIP 2634 kb) [file 12881_2018_626_MOESM2_ESM.zip › Fig S3R3.tif]
